# CDC Grand Rounds: Addressing Health Disparities in Early Childhood

**DOI:** 10.15585/mmwr.mm6629a1

**Published:** 2017-07-28

**Authors:** Lara R. Robinson, Rebecca H. Bitsko, Ross A. Thompson, Paul H. Dworkin, Mary Ann McCabe, Georgina Peacock, Phoebe G. Thorpe

**Affiliations:** ^1^Division of Human Development and Disability, National Center on Birth Defects and Developmental Disabilities, CDC; ^2^Department of Psychology, University of California, Davis; ^3^Department of Pediatrics, University of Connecticut School of Medicine;^4^Department of Pediatrics, George Washington University School of Medicine, Washington, DC; ^5^Office of the Associate Director of Science, Office of the Director, CDC.

Research suggests that many disparities in overall health and well-being are rooted in early childhood (*1*,*2*). Stressors in early childhood can disrupt neurologic, metabolic, and immunologic systems, leading to poorer developmental outcomes (*1*). However, consistent, responsive caregiving relationships and supportive community and health care environments promote an optimal trajectory (*3*,*4*). The first 8 years of a child’s life build a foundation for future health and life success (*5*–*7*). Thus, the cumulative and lifelong impact of early experiences, both positive and negative, on a child’s development can be profound. Although the health, social service, and education systems that serve young children and their families and communities provide opportunities to support responsive relationships and environments, efforts by these systems are often fragmented because of restrictions that limit the age groups they can serve and types of services they can provide. Integrating relationship-based prevention and intervention services for children early in life, when the brain is developing most rapidly, can optimize developmental trajectories (*4*,*7*). By promoting collaboration and data-driven intervention activities, public health can play a critical role in both the identification of at-risk children and the integration of systems that can support healthy development. These efforts can address disparities by reducing barriers that might prevent children from reaching their full potential.

## Developmental Trajectories

Healthy child development includes not only physical developmental domains but also emotional, behavioral, cognitive, language, and general learning competencies. The human brain undergoes rapid growth during childhood, driven in part by a child’s acquisition and integration of skills across many developmental domains. Development in all domains is finely integrated across neural circuitry, allowing for more complex learning and tasks over time (*8*). Skill acquisition depends on children being ready to learn and can be envisioned as a developmental trajectory.

Exposure to adversity and stressors such as poverty, lack of safety and stability in the home environment, and lack of access to quality early education can negatively affect a child’s development (*1*,*2*). These exposures can lead to an “at-risk or vulnerable” trajectory and in severe cases, a “delayed or disordered” trajectory (*5*). Conversely, protective factors provided in a child’s home or community environment, such as consistent and responsive caregiving relationships and coordinated health care and other services, can reduce and even ameliorate the impact of adverse circumstances, allowing children to reach or return to a healthy trajectory (*2*,*5*).

Chronic stressors in early childhood, such as poverty, can have cumulative lifetime effects on learning, earnings, and health (*3*). Language differences associated with socioeconomic status have been documented as early as age 18 months (*9*). Vocabulary skills by age 3 years predict third grade reading, which in turn predicts high school graduation rates (*10*–*12*). High school graduates achieve increased earning potential and are less likely to have chronic diseases, such as diabetes, chronic pain, and symptoms of mental disorders than are non-graduates (*13*). High school graduates are also more likely to report good health and visit a health professional, important markers of positive health outcomes (*13*).

## Identifying Vulnerable Children and Informing Action

Screening, early identification, and linkage to services can prevent vulnerable children (i.e., children at risk for or with a developmental delay) from progressing to levels of higher risk (*14*). For disadvantaged groups, early intervention can yield the greatest social and economic returns (*15*). For example, an economic analysis of two similar early childhood interventions for socioeconomically disadvantaged children, Carolina Abecedarian Project and the Carolina Approach to Responsive Education, identified a 7.3 benefit/cost ratio and a 13.7% rate of return per annum when examining the long-term health, crime reduction, educational, and employment benefits of program participation (*15*).

Public health surveillance data characterize population-level impacts and can be used to inform public health action. For example, recent analyses identified treatment patterns for young children with attention-deficit/hyperactivity disorder that were not aligned with the American Academy of Pediatrics’ (AAP) recommendations (*16*). These data have led to collaborations to 1) increase awareness of recommendations for behavior therapy before medication for preschool children, 2) increase available behavioral therapy options for providers and families, and 3) inform state and local decision-makers about best practices (*16*). Surveillance data continue to inform and monitor the impact of these collaborations and other early childhood initiatives.

Screening measures inclusive of social determinants of health provide opportunities for strengthening protective factors through family, community, and health care connections (*3*). Public health activities to improve early detection and referral to treatment include the Early Hearing Detection and Intervention* programs to identify hearing loss in infants; online tools developed by CDC and AAP for identifying motor delays^†^; and Learn the Signs. Act Early^§^ for children with or at risk for developmental disabilities. These tools leverage state, provider, and family-level actions to reduce the time to diagnosis and initiation of services.

## Integrating Support Services for Vulnerable Children and Their Families

A large number of service agencies work to support optimal child development, but many have specific age requirements (e.g., early intervention, preschool, or school age), or provide specific types of services (e.g., developmental, health, social welfare, or educational). Too often, vulnerable children are identified but do not meet strict criteria for services of the agencies contacted, leaving them without needed services. An example of a program that has reduced service gaps by integrating available services for children is Help Me Grow.^¶^ Help Me Grow serves as a centralized point of entry for both state- and community-based services where families of vulnerable children are matched to service agencies that offer the support they need (*14*). Through a single information line, vulnerable children who are likely to meet eligibility criteria are linked to one or more publicly funded early intervention services, preschool special education services, and interventions for children with special health care needs. Vulnerable children at risk because of environmental or biologic factors, but who do not meet eligibility requirements for the described services are linked to other community-based programs and services through Help Me Grow. In 2015 alone, Help Me Grow served 42,511 children and their families. Promising evaluation results have led some states to embed the Help Me Grow model within various federal initiatives, including the Health Resources and Services Administration’s Maternal, Infant, and Early Childhood Home Visiting and Early Childhood Comprehensive Systems and the Substance Abuse and Mental Health Services Administration’s Project LAUNCH (Linking Actions for Unmet Needs in Children’s Health) program.

## Integrating Behavioral and Physical Health

Behavioral health services can promote the health and development of children when high-quality services can be accessed by the children who need them (*17*). Nationally representative data from 2011–2012 suggest that 15% of U.S. children aged 2–8 years have a parent-reported mental, behavioral, or developmental disorder (*18*), and children living in small rural areas have a higher prevalence (19%) than children living in urban areas (15%) (*19*). In 2012, nearly $14 billion in medical expenditures for mental disorders among children were spent across all payment types (private insurance, public insurance, out of pocket, and other); these costs were higher than those for any other health condition (e.g., chronic obstructive pulmonary disease and asthma, trauma-related conditions, and acute respiratory infections).** However, only an estimated 20% of children and youth with behavioral problems receive mental health services (*17*). In particular, children in rural communities often have less access to early childhood interventions and behavioral health care services highlighting the need for behavioral health care in alternative settings and coordinated care solutions (*20*).

Mental, behavioral, and developmental disorders in young children have been associated with potentially modifiable family, community, and health care factors (*18*,*19*). Two-generation approaches that support the health, educational achievement, economic self-sufficiency, and wellbeing of both children and their caregivers have indicated some beneficial effects on early childhood literacy and language development (*3*,*7*). Within primary care, screening and referral to appropriate services for maternal depression can support the parent-child relationship and enhance both child and maternal health (*3*). For children facing circumstances that put them at risk, such as poverty, enhancing these maternal-child protective factors might be particularly important for reducing the negative effects of stressors on long-term child health (*3*). Furthermore, pediatric primary care can expand beyond anticipatory guidance by promoting protective factors and resiliency through evidence-based interventions that address parental self-care, positive parenting strategies, and parent-child relationship building (*3*,*7*). By coordinating and integrating care across medical systems and community providers, the prevention- and patient-focused medical home (family-centered coordinated primary care) model promotes both behavioral and physical health.

## Promoting Supportive Relationships Across Multiple Contexts

Early childhood objectives outlined in *Healthy People 2020*^††^ highlight the need to support parents and caregivers, create supportive communities, increase access to high-quality health care, and increase the proportion of children ready for school in all domains of healthy development. Programs that create connections across the early learning and home environments by supporting family engagement in learning have demonstrated positive impacts on young children’s academic success and development (*7*,*8*). However, gaps exist in access to high quality early care and education, training, and evidence-based resources to support family engagement partnerships (*7*,*8*). A 2016 AAP policy statement aimed at ameliorating the health and developmental impacts of poverty describes the importance of effective interventions and strategies focused on economic aid, access to comprehensive care coordination, early care and education, early identification of children and families in need of services, and promotion of protective factors through family support programs (*3*). The common thread for these approaches is the focus on both risk factors and protective factors for the entire family across multiple systems, not simply on the child with an identified condition in a single context.

## Importance of Integration and Collaboration

Early childhood represents a period of growth that lays the foundation for successful learning, development, and health; disparities emerge early and widen over time (*6*). Intervening in early childhood can prevent the development of diseases and disorders among at-risk and vulnerable children but will require collaboration. Strategies that foster consistent and responsive caregiving relationships and supportive environments can improve outcomes for both parent and child (*7*). Parents and early care providers can work together to provide the responsive interactions and consistent environments that nurture the development of young children. Practitioners can screen and identify children early, promote family strengths, and refer to services before risks progress. States and communities can use surveillance data to drive action around early childhood investments. Partners within public health can use data-informed approaches to prevent health disparities by facilitating service linkages across health, social, and educational systems. Timely referral and better integrated services might help children at low or moderate risk reach their full potential by returning to healthy developmental trajectories.

## Footnotes

* https://www.cdc.gov/ncbddd/hearingloss/ehdi-programs.html.

† http://motordelay.aap.org.

§ https://www.cdc.gov/ncbddd/actearly/index.html.

¶ https://helpmegrownational.org.

** https://meps.ahrq.gov/data_files/publications/st472/stat472.shtml.

†† https://www.healthypeople.gov/node/3498/objectives#4816.
